# Efficacy of Preemptive Analgesia with Amantadine for Controlling Postoperative Pain in Cats Undergoing Ovariohysterectomy

**DOI:** 10.3390/ani14040643

**Published:** 2024-02-17

**Authors:** Paula Elisa Brandão Guedes, Taísa Miranda Pinto, Janaína Maria Xavier Corrêa, Raquel Vieira Niella, Carolina Moreira dos Anjos, Jéssica Natália Silva de Oliveira, Claire Souza da Costa Marques, Sophia Saraiva de Souza, Elisângela Barboza da Silva, Mário Sérgio Lima de Lavor

**Affiliations:** 1 Postgraduate Program in Animal Science, State University of Santa Cruz (UESC), Ilhéus 45662-900, BA, Brazil; paulaebg@gmail.com (P.E.B.G.); taisa_pink@hotmail.com (T.M.P.); janainaxaviercorrea@gmail.com (J.M.X.C.); raquelniella@hotmail.com (R.V.N.); carolinamanjos@hotmail.com (C.M.d.A.); jeoliveira.rp25@gmail.com (J.N.S.d.O.); clairefaculdade@gmail.com (C.S.d.C.M.); sophiasaraivas@gmail.com (S.S.d.S.); 2 Department of Agricultural and Environmental Sciences, State University of Santa Cruz (UESC), Ilhéus 45662-900, BA, Brazil; ebsilva@uesc.br

**Keywords:** acute pain, analgesics, cats, NMDA antagonist

## Abstract

**Simple Summary:**

Amantadine, a drug initially used as an antiviral agent to treat the influenza A virus (though currently, its main application is in the treatment of Parkinson’s disease) has also been used to control pain due to its mechanism of action: exerting non-competitive antagonism N-methyl-D-aspartate (NMDA) glutamatergic receptors, which participate in the neurophysiological project of pain via the inhibition of central sensitization. We hope that our study makes a significant contribution to the literature because the preemptive oral administration of amantadine at a dose of 5 mg/kg resulted in superior postsurgical pain control. In addition, the administration of amantadine did not result in cardiovascular or respiratory alterations or adverse effects during the intraoperative period of OVH in the evaluated cats.

**Abstract:**

This study aimed to evaluate the effect of the preemptive administration of amantadine on postoperative analgesia in cats undergoing ovariohysterectomy and its influence on the physiological parameters. Twenty healthy domestic cats scheduled to undergo ovariohysterectomy at the Santa Cruz State University, Ilhéus, were divided into two groups: the control group (Group C; *n* = 10) and the amantadine group (Group A; *n* = 10). The cats in Group C received placebo capsules 30 min prior to the standard anesthetic protocol, whereas those in Group A received 5 mg/kg of amantadine orally 30 min prior to the standard anesthetic protocol. Postoperative pain was assessed using the visual analog scale and the UNESP-Botucatu multidimensional scale for the evaluation of postoperative pain in cats. The administration of amantadine had no effect on the physiological parameters evaluated. The pain scores in Group A were lower than those in Group C, indicating that the frequency of rescue analgesic administration cats in Group A was lower. That way, preemptive oral administration of amantadine at a dose of 5 mg/kg was effective at controlling postoperative pain in cats undergoing ovariohysterectomy. Moreover, no adverse effects or alterations in the physiological patterns were observed in the treated animals.

## 1. Introduction

The International Association for the Study of Pain (IASP) defines pain as “An unpleasant sensory and emotional experience associated with, or resembling that associated with, actual or potential tissue damage.” [1]. Pain in response to tissue damage occurs through the release of chemical mediators, mechanical and thermal stimuli that stimulate afferent fiber nociceptors [2,3]. Acute pain is observed in patients undergoing surgical procedures, especially in procedures wherein visceral organs are manipulated, such as ovariohysterectomy (OVH) [4].

Surgical procedures are commonly performed in veterinary medicine for the sterilization of companion animals [5], population control of stray animals [6], and the treatment of uterine infections [7] and reproductive tract cysts [8]. OVH is an effective and safe contraceptive method that is recommended before or at six months of age to reduce the risk of mammary gland adenocarcinoma [9].

Several clinical and experimental studies have been conducted on the management of intraoperative and postoperative pain through the use of effective anesthetic protocols that include the preemptive and intraoperative administration of different classes of drugs, such as amantadine [10,11,12]. OVH is a widely used model for these studies, especially in cats, as it is the most commonly performed surgical procedure in this species [13,14].

Optimal pain management in veterinary practice not only ensures the welfare and comfort of animal patients but also aligns with ethical imperatives and advances in veterinary care. The imperative to address postoperative pain in feline patients, particularly after OVH, arises not only from an ethical standpoint but also due to its impact on the animal’s recovery, behavior, and overall well-being. Unmanaged or inadequately managed pain not only induces distress but can potentially lead to prolonged recovery periods, altered physiology, and can adversely affect the animal’s behavior and immune response [15].

Amantadine is an antiviral agent [16]; however, it has a non-competitive antagonistic action on the glutamatergic receptors of the N-methyl-D-aspartate (NMDA) type, which is involved in the neurophysiology of pain [17,18]. This preemptive blockade of NMDA receptors not only disrupts the initial transmission of nociceptive signals but also impedes the subsequent neuroplastic changes, leading to the hypersensitization of pain pathways. Furthermore, recent investigations [16,17] have elucidated amantadine’s additional modulation of downstream intracellular signaling pathways involved in synaptic plasticity, underscoring its multifaceted role in curtailing the development of hyperexcitability within the nociceptive circuitry. The preventive action of amantadine on NMDA receptors stands as a promising strategy to halt or minimize central sensitization, offering a potential avenue for preemptive intervention in acute pain management [19,20]. Thus, amantadine has been used successfully in the management of chronic pain (neuropathic and inflammatory) and acute pain [12,19,20,21,22,23,24].

Human studies have shown that preoperative administration of oral amantadine reduces the postoperative morphine requirement in patients undergoing radical prostatectomy [23]. Mitch et al. [25] concluded that the combination of amantadine and non-steroidal anti-inflammatory drugs, as part of a multimodal analgesic treatment, improves physical activity in dogs with refractory osteoarthritis. Other studies have demonstrated that the preemptive administration of amantadine reduced the analgesic requirement in dogs undergoing OVH [12] and was effective as a multimodal therapy for the treatment of neuropathic pain in dogs [20].

Snijdelaar and Koren [19] demonstrated that the oral administration of 5 mg/kg of amantadine for three weeks resulted in satisfactory results and an improvement in quality of life in cats with osteoarthritis. However, data on the use of amantadine for the management of acute postoperative pain in cats are not available.

The choice of amantadine was based on the results of studies indicating its effectiveness in the management of pain in dogs [12,21,25], humans [22], and cats [19,21]. The pharmacokinetics of amantadine in the cat have been published, showing systemic availability and half-life after oral administration of 130 ± 11 (86–160)% and 324 ± 41 (277–381) min, respectively, which makes it favorable due to the prolonged time of action in the animal’s body [26]. Additionally, it was able to improve owner-identified impaired mobility and owner-perceived quality of life in cats with osteoarthritis [21].

Amantadine may aid in the management of acute postoperative pain of visceral origin by inhibiting nociceptive transduction, preventing central sensitization, and sensitizing peripheral nerves, promoting adequate analgesia in cats undergoing OVH [27,28]. Several studies have been conducted to evaluate the efficacy of amantadine. However, to date, no published data on its analgesic action in cats undergoing OVH are available. Therefore, this study aimed to evaluate the effect of the preemptive administration of amantadine on postoperative analgesia in cats undergoing OVH and its influence on the physiological parameters of the treated animals. By focusing on refining analgesic strategies, this study endeavors not only to alleviate immediate postoperative discomfort but also to contribute to enhancing the recovery and overall welfare of feline patients undergoing OVH, contributing to the advancement of feline surgical care standards.

## 2. Materials and Methods

### 2.1. Animals and Ethical Considerations

This study was approved by the Ethics Committee on the Use of Animals of the Universidade Estadual de Santa Cruz (CEUA-UESC) (protocol number: 004/17) and was conducted in accordance with the Ethical Principles of Animal Experimentation.

Twenty docile domestic cats (age: 1–4 years; weight: 2.5–3.3 kg) with no specific breeds noted who were scheduled to undergo elective OVH at the Veterinary Hospital of UESC were included in this study. After the experimental design was explained, informed consent was obtained from the owners prior to the inclusion of the cats. The animals subsequently underwent clinical examinations. Blood tests were performed to evaluate the complete blood count. Ultrasonography was also performed if pregnancy was suspected. Clinically healthy cats with hematological parameters within the normal range that were not pregnant were included in the study.

### 2.2. Experimental Groups and Anesthetic–Surgical Protocol

The cats were divided into two groups using block randomization, with ten animals in each group, so that the ages were divided in a similar way. The cats enrolled in the control group (Group C) received placebo capsules 30 min prior to the administration of preanesthetic medication (PAM). The cats enrolled in the amantadine group (Group A) received 5 mg/kg of amantadine capsules 30 min prior to the administration of PAM.

The animals were observed for 30 min after the administration of the capsule (placebo or amantadine) to evaluate the presence of vomiting, excitement, sedation, and pupillary dilation.

As a standard preanesthetic procedure, the patients were fasted for 12 h prior to the surgery. The consumption of water was restricted for 2 h prior to the surgery. All cats underwent a preanesthetic evaluation 1 h before the start of the surgery (M0) for the evaluation of the following parameters: heart rate (HR), respiratory rate (RR), and body temperature (T °C). The surgeon and the evaluators who performed the pre- and postoperative evaluations were blinded to the group allocation.

As part of the standard anesthetic protocol, the cats in both groups received acepromazine (0.03 mg/kg; 0.2%; Acepran^®^, Vetnil, Brazil) and meperidine (3 mg/kg; pethidine hydrochloride, 50 mg/mL; União Química, Brazil) intramuscularly (IM) as PAM. The cephalic vein was catheterized using a 24-gauge catheter (Radiopaque Safelet Catheter; Nipro Medical Corporation Produtos Médicos Ltd., São Paulo, Brazil) 20 min after administering PAM, and the animals received Lactated Ringer’s solution at a rate of 6 mL/kg/h until the end of the surgical procedure. For anesthetic induction, 5 mg/kg of propofol was administered intravenously (IV). Anesthesia was maintained with isoflurane in 100% oxygen (300 mL/kg/min) using a non-rebreathing system (Mapleson Breathing Systems, Jackson Rees) after endotracheal intubation. Isoflurane was administered at a concentration of 1.0 V% initially, and the concentration was adjusted according to the autonomic system response (HR, RR, and SBP) to the surgical stimulus. The concentration was increased to 1.5 V% if an increase of 20% was observed in the HR or systolic blood pressure (SBP).

After anesthetic induction, sodium cephalothin (30 mg/kg, IV) (Ceflen; Agila, Brazil) was administered as antibiotic therapy. Surgical antisepsis was subsequently performed, followed by OVH, which was performed by the same surgeon in all animals through a ventral median incision of approximately 5 cm to access the uterine horns. Polyglactin 910 wire of 2-0 gauge was used to ligate the ovarian pedicles of the uterine stump and reduce the subcutaneous dead space, and 2-0 polyamide thread was used to suture the abdominal wall and skin. The stitches were removed 10 days after the procedure.

### 2.3. Intraoperative Evaluation of Physiological Parameters

The physiological parameters were evaluated intraoperatively at the following time points: M1, before the beginning of the surgical procedure; M2, after the incision of the *linea alba*; M3, after clamping the first pedicle; M4, after clamping the second pedicle; M5, after the ligation of the uterine stump; M6, muscle suturing; and M7, upon completion of the surgery.

The following variables were monitored using a multiparametric monitor during these periods: HR, RR, oxyhemoglobin saturation (SpO_2_), end-tidal carbon dioxide (ETCO_2_), end-tidal isoflurane concentration (Etiso), esophageal temperature (T °C), and SBP. SBP was measured via an indirect method using ultrasonic Doppler (Portable Vascular Doppler Medmega DV 610) and a cuff measuring 30–40% of the circumference of the animal’s radioulnar region.

### 2.4. Pain Assessment

The patients were observed after emergence from anesthesia. Postoperative pain was evaluated at the following time points using the VAS and the UNESP-Botucatu multidimensional scale: one (T1), two (T2), three (T3), four (T4), five (T5), and six (T6) hours after the end of the surgery.

The VAS is a semi-objective scoring system for evaluating pain, with values ranging from 0 (no pain) to 100 mm (greatest possible pain) [26].

The UNESP-Botucatu multidimensional scale is a scale for assessing postoperative pain in cats, wherein the scores vary from 0 (no pain) to 30 points (maximum pain) [27]. It is based on observations of behavior, interaction, and physical assessment of the patient. During the 6 h postoperative evaluation, the animals were monitored for signs of salivation and/or vomiting.

The VAS was completed prior to the Multidimensional Scale to ensure that its scores were not influenced by the questionnaire responses.

### 2.5. Rescue Analgesia

Rescue analgesics were administered when scores equivalent to ≥11 points [27] on the UNESP-Botucatu Multidimensional Scale were observed and/or the VAS value was ≥40 mm [28]. The animals received 1 mg/kg IM of meperidine in these cases.

### 2.6. Postsurgical Therapy

After six hours of pain assessment, the cats returned home with their owners and all animals received 4 mg/kg of IM tramadol (Tramadol hydrochloride, 50 mg/mL, União Química, Brazil), 0.2 mg/kg of IM meloxicam (0.2% Maxicam; Ourofino, Brazil), and 25 mg/kg of IM dipyrone (D-500, sodium dipyrone, 500 mg/mL, Zoetis, Brazil) after the final evaluation. For complementary analgesia, 0.1 mg/kg of meloxicam was administered orally every 24 h for 4 days.

### 2.7. Statistical Analysis

The data were analyzed using Prism 5 (GraphPad Software 8.0, La Jolla, CA, USA) for Windows 10 (Microsoft Corporation, Redmond, WA, USA). The sample size was based on previous experiments, wherein sample calculation considered the standard deviation, the difference between the mean obtained in the sample and the true mean, and the critical values of the Student’s t-distribution [29]. The Kolmogorov–Smirnov test was used to assess the normality of the data. The data of all parameters (HR, SBP, RR, SpO_2_, EtCO_2_, Etiso, and T °C) showed normal distribution and were subjected to analysis of variance. The means were compared using the Bonferroni test. The Mann–Whitney and Friedman tests were performed to compare the non-parametric data between groups, followed by Dunn’s post hoc test. Fisher’s exact test analysis was performed on rescue analgesia in postoperative evaluations. The significance level was set at 5%.

## 3. Results

No significant differences were observed between Groups C and A in terms of weight, propofol dosage, operative time, or extubation time (Table 1).

No changes in behavior or vomiting, excitement, sedation, or pupillary dilation were observed after the administration of the placebo or amantadine capsule.

### 3.1. Assessment of the Physiological Parameters

The comparison between the means of the evaluated parameters revealed no statistically significant differences between the groups at any time point (Table 2).

### 3.2. Pain Assessment

**Figure 1:**
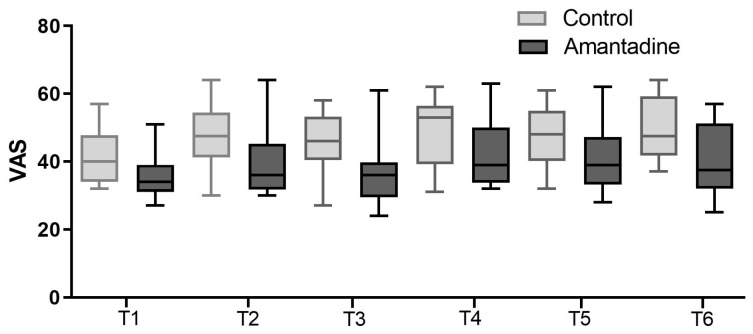
Box plot representing the pain score on the visual analog scale for the control (C) and amantadine (A) groups in the evaluations corresponding to one (T1), two (T2), three (T3), four (T4), five (T5), and six (T6) hours after surgery. Mann–Whitney test (*p* < 0.05).

**Figure 2:**
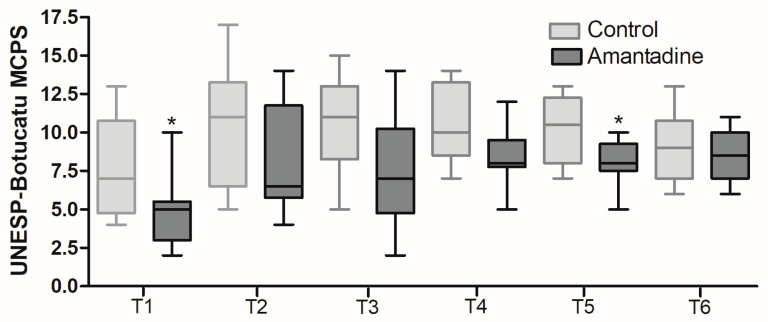
Pain score of the UNESP-Botucatu Multidimensional Scale. * Box plot representing the pain score of the UNESP-Botucatu Multidimensional Scale for assessing postoperative pain in cats for the control (C) and amantadine (A) groups, one (T1), two (T2), three (T3), four (T4), five (T5), and six (T6) hours after surgery. Mann–Whitney test, T1 (*p* = 0.0428) and T5 (*p* = 0.0308).

There were no statistical differences in the VAS (Figure 1); however, the UNESP-Botucatu multidimensional scale scores were lower in Group A (Figure 2), with significant differences of *p* = 0.0043 and *p* = 0.0260 (Mann–Whitney), respectively.

### 3.3. Rescue Analgesics

The frequency of administration of rescue analgesics in Group C was higher than in Group A and, considering the percentage of rescue analgesia in postoperative evaluations, this difference was significant (*p* < 0.05) (Table 3).

## 4. Discussion

Amantadine was administered as a preemptive analgesic in this study, as compared with postoperative administration of analgesic drugs alone, the preemptive administration of analgesics results in a significant reduction in hyperalgesia [30]. In preemptive analgesia, analgesic drugs are administered before tissue injury to inhibit the process of peripheral and central sensitization, thereby minimizing postoperative pain and the recovery period [31,32].

Amantadine was administered orally at a dose of 5 mg/kg [19,21,26] 30 min before PAM, based on pharmacokinetics. The maximum plasma concentration in cats is attained 1.5–5 h after oral administration [26], which is a sufficient duration to complete OVH. In addition, the six-hour postsurgical period adopted for analgesic evaluation in this study was based on the half-life of amantadine in cats, which ranges from 4.6 to 6.35 h [26].

The absence of adverse effects in this study corroborates the results of other studies, indicating the safety of the drug at the administered dose [12,21,33]. An increase in HR was observed at M3 and M4 in both groups, which was attributed to the response of the autonomic nervous system to the greatest painful stimulus at these time points (clamping of the ovarian pedicles). A similar finding was observed in a study by Silva [34], which evaluated pain in dogs undergoing OVH. These results are consistent with those of previous reports, which demonstrated that the administration of amantadine had no effect on the physiological parameters at the administered dose [12].

No differences were observed between the SpO_2_ and ETCO_2_ of the two groups, indicating that amantadine had no effect on the respiratory parameters of the evaluated cats, which is consistent with the results of the study by Niella et al. [11]. According to the literature, the level of normality of SpO_2_ in animals receiving 100% oxygen ranges from 98 to 100%, indicating that all cats included in this study had values within the normal range without affecting oxygenation. The same was observed with regard to ETCO_2_, as both groups maintained the parameter within the normal range for the species, although inhalational anesthetics may promote an increase in ETCO_2_ values as a result of respiratory depression [35]. SpO_2_ and ETCO_2_ were not recorded at baseline (M0) due to the difficulty in completing the evaluation using the equipment, as the animals were conscious.

Regarding the evaluation of Etiso, the concentration necessary to maintain the animals in the surgical plane was used initially, according to Guedel’s criteria. A reduction in Etiso was observed in both groups at the last time point (M7), and a smaller amount of inhalational anesthetic was required to maintain the animals in an adequate anesthetic plane due to the reduction in surgical stimuli.

Studies in human medicine have shown that orally administering amantadine in the preoperative period results in a reduction in the concentrations of isoflurane during the surgical period [36]. However, in this study, as observed in the study conducted by Niella et al. [11], amantadine did not affect this parameter.

A reduction in body temperature was observed in both groups during the evaluation [37]. The reduction in T °C was attributed to general anesthesia due to the reduction in the metabolic activities and thermoregulatory hypothalamic mechanisms caused by the antisepsis process, opening of the abdominal cavity, and fluid therapy at room temperature [38]. This finding was also reported by Silva et al. [34] during the intraoperative period of dogs undergoing OVH.

According to Paepe et al. [33], the SBP in conscious cats measured using Doppler ranges from 80 to 160 mmHg. However, in anesthetized patients, this value may be lower due to the adverse effects caused by general anesthetics [39,40], as observed in this study. Patients with SBP values below 80–90 mmHg during anesthesia were considered hypotensive [41]. Borderline or lower than the recommended values were noted at M1 and M2 in both groups. The reduction in SBP at these times may have occurred due to the use of acepromazine, which acts as a pharmacological potentiator of general anesthetics. Its main effect is alpha-adrenergic blockade, which causes dose-dependent hypotension [40,41]. When used for anesthetic induction, propofol can also lead to a decrease in SBP and a slight increase in heart rate due to the decrease in sympathetic activity, as reported in previous studies [41]. However, an increase in SBP and HR was observed at M3 and M4 in both groups owing to the clamping of the ovarian pedicles, as reported by Silva et al. [34].

Using standardized and validated pain assessment tools is important because it produces more consistent and accurate results. The Botucatu scale, with established reliability, showed a significant difference and the number of rescues was significantly lower in Group A than in Group C, which indicates an indication for amantadine in reducing postoperative pain in cats undergoing OVH, as observed in dogs [11].

Furthermore, the availability of a criterion for rescue analgesia is a valuable tool in assisting the observer making decisions about analgesic therapy. Together with pain scores, this may also provide an important measure of the efficacy of analgesic therapy [23]. It was observed that rescue analgesics were administered less frequently in Group A than in Group C, indicating a significant reduction in acute postoperative pain. The preemptive administration of amantadine blocks the NMDA receptors, thereby impeding the excitatory action of glutamate on these receptors and consequently preventing or minimizing central sensitization, a pivotal mechanism in the development and perpetuation of acute pain. By impeding the excitatory action of glutamate on NMDA receptors, amantadine acts as a molecular regulator, preventing the amplification and propagation of pain signals within the central nervous system [16,17].

The results of this study corroborate those of previous studies wherein the preoperative administration of amantadine reduced the requirement for analgesic rescue after anterior cruciate ligament reconstruction surgery in humans [40]. When compared to the placebo group in abdominoplasty surgery in women [36] and dogs undergoing OVH, the cats who received preoperative amantadine required fewer analgesic rescues than those in the control group during the postoperative period [12].

This study demonstrates the efficacy of the preoperative administration of amantadine in the prevention or reduction of acute postoperative pain. The findings of this study encourage further research on the management of acute postoperative pain in veterinary patients.

## 5. Conclusions

Preemptive oral administration of amantadine at a dose of 5 mg/kg resulted in superior postsurgical pain control. In addition, the administration of amantadine did not result in cardiovascular or respiratory alterations or adverse effects during the intraoperative period of OVH in the evaluated cats.

## Figures and Tables

**Table 1:** Mean and standard deviation of weight, propofol dose, surgery time, and extubation time of cats undergoing OVH: control and treatment group (C and A).

Variables	C	A
Weight (kg)	2.8 ± 0.44	2.7 ± 0.38
Dose of Propofol (mg/kg)	5.2 ± 0.63	5.6 ± 1.24
Surgery time (min)	25.3 ± 4.27	24.6 ± 1.17
Extubation time (min)	7.2 ± 1.32	7 ± 1.33

**Table 2:** Variables evaluated in cats studied: control and treatment group (C and A), at moments M0 to M7.

Variables	Groups	M0	M1	M2	M3	M4	M5	M6	M7
HR (bpm)	C	209 ± 4.87	157 ± 5.70	153 ± 7.05	192 ± 9.13	184 ± 9.59	173 ± 8.02	163 ± 122.5	163 ± 7.43
	A	205 ± 5.84	154 ± 5.95	145 ± 8.76	170 ± 8.68	175 ± 7.23	167 ± 6.53	162 ± 5.80	158 ± 7.42
RR(rpm)	C	63 ± 2.42	25 ± 1.97	26 ± 3.02	29 ± 3.04	29 ± 3.26	29 ± 3.02	25 ± 3.31	24 ± 2.62
	A	61 ± 1.52	22 ± 2.05	20 ± 3.04	21 ± 2.85	22 ± 3.17	21 ± 3.44	19 ± 2.51	21 ± 2.92
SpO_2_ (%)	C	-	98.3 ± 0.59	98.4 ± 0.61	99.1 ± 0.50	98.8 ± 0.48	98.9 ± 0.52	99.2 ± 0.48	99.7 ± 0.15
	A	-	98.8 ± 0.51	98.3 ± 0.71	99.5 ± 0.22	99 ± 0.25	98.7 ± 0.42	98.8 ± 0.61	98.5 ± 0.67
ETCO_2_	C	-	32.0 ± 2.74	30.6 ± 2.61	31.4 ± 1.99	34.2 ± 2.1	34.4 ± 2.00	34.8 ± 2.07	31.4 ± 2.17
	A	-	32.6 ± 1.99	36 ± 2.38	39.6 ± 2.38	40.8 ± 2.49	39.8 ± 2.49	36.8 ± 2.25	38.8 ± 2.93
Etiso(%)	C	-	1.43 ± 0.08	1.32 ± 0.06	1.39 ± 0.08	1.46 ± 0.07	1.48 ± 0.12	1.27 ± 0.07	0.71 ± 0.08
	A	-	1.43 ± 0.04	1.36 ± 0.06	1.33 ± 0.04	1.35 ± 0.05	1.4 ± 0.06	1.29 ± 0.06	0.79 ± 0.05
T(°C)	C	38.67 ± 0.10	37.86 ± 0.17	37.43 ± 0.19	37.15 ± 0.20	36.9 ± 0.22	36.71 ± 0.26	36.52 ± 0.27	36.32 ± 1.33
	A	38.66 ± 0.07	37.55 ± 0.16	37.09 ± 0.15	36.93 ± 0.14	36.65 ± 0.20	36.45 ± 0.27	36.22 ± 1.36	35.95 ± 1.40
SBP (mmHg)	C	-	65.02 ± 3.32	79.1 ± 5.53	112.5 ± 14.17	121.4 ± 11.22	99.1 ± 5.33	93.4 ± 4.53	99.8 ± 2.23
	A	-	80 ± 6.29	83.3 ± 5.00	114.9 ± 11.88	126.4 ± 11.29	106.4 ± 8.01	96.9 ± 7.01	107.3 ± 11.07

The results are expressed as means and standard error of mean. M0: one hour before the start of the experiment, M1: before the beginning of the surgical procedure, M2: after incision of the *linea alba*, M3: after clamping the first pedicle, M4: after clamping the second pedicle, M5: after ligation of the uterine stump, M6: muscle suture, M7: end of surgery.

**Table 3:** Number of cats undergoing OVH that received analgesic rescue, control group (C) and amantadine group (A), in the moments of postoperative evaluation T1 to T6.

	T1	T2	T3	T4	T5	T6	Total
C	2	4	0	1	1	0	8/10 (80%) *
A	0	3	0	1	0	0	4/10 (40%)

* Significant difference between groups.

## Data Availability

The datasets used and/or analyzed during the current study are available from the corresponding author on reasonable request.
